# Massive Left Atrial Thrombus in Two Patients with Heparin-Induced Thrombocytopenia Type II after Cardiac Surgery

**DOI:** 10.1055/s-0038-1672188

**Published:** 2018-09-26

**Authors:** Yuriy Mandryk, Markus Czesla, Christian Mogilansky, Kristina Stefkova, Aloys Drees, Parwis Massoudy

**Affiliations:** 1 Department of Cardiac Surgery, Klinikum Passau, Passau, Germany; 2 Department for Laboratory Medicine, Klinikum Passau, Passau, Germany

**Keywords:** heparin-induced thrombocytopenia, argatroban, mitral valve replacement, endocardial cryoablation

## Abstract

Heparin-induced thrombocytopenia type II (HIT type II) can have devastating consequences in cardiac surgical patients. We report two cases of massive left atrial thrombus after mitral valve replacement and endocardial cryoablation in patients with HIT type II.

## Clinical Summary

**Fig. 1:**
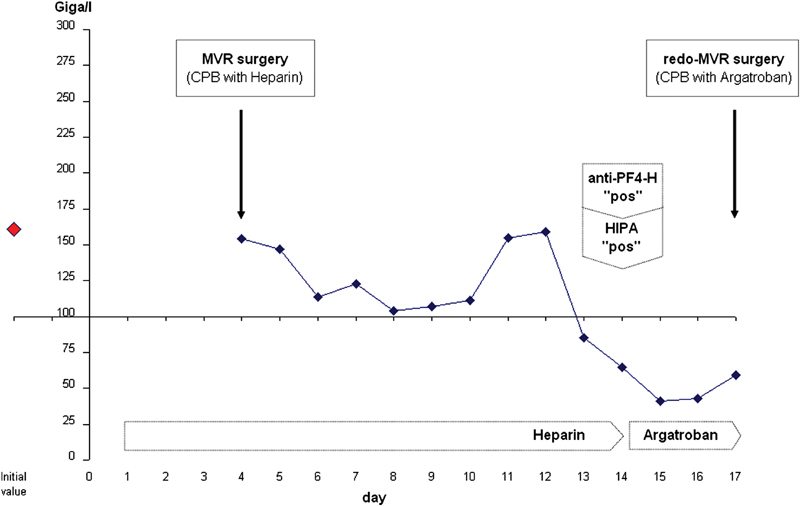
Thrombocyte count (y-axis) in patient A with early left atrial thrombus formation 10 days after surgery. x-axis represents time line. Unfractioned heparin was started on the day of hospital admission as an intravenous application, because of atrial fibrillation. In the preoperative period, target partial thromboplastin time (PTT) was 60–80 seconds; intraoperatively, target ACT was 400 seconds; and postoperatively, target PTT was again 60–80 seconds. Time of PF4-H IgG-specific ELISA and HIPA tests and time of redo-MVR surgery are indicated by arrows. Result for ELISA was 2.09 OD, for HIPA 4 of 4 cells (details in Clinical Summary). Argatroban infusion was started on day 13 after MVR surgery, with a target value of 1.0 ng/mL.

**Fig. 2:**
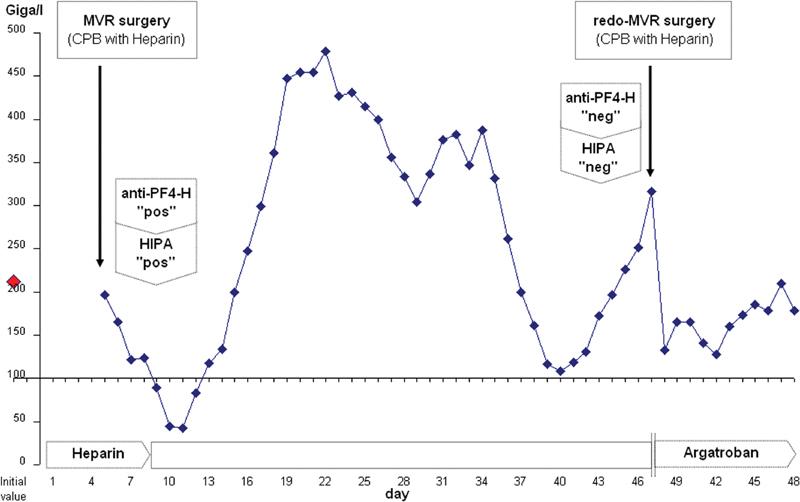
Thrombocyte count (y-axis) in patient B with delayed left atrial thrombus formation 5 weeks after surgery. x-axis represents timeline. Unfractioned heparin was started on the day of hospital admission as an intravenous application, because of atrial fibrillation. In the preoperative period, target PTT was 60–80 seconds; intraoperatively, target ACT was 400 seconds; and postoperatively, target PTT was again 60–80 seconds. Time of PF4-H IgG-specific ELISA and HIPA tests and time of redo-MVR surgery are indicated by arrows. Result for ELISA was 2.04 OD, for HIPA 4 of 4 cells (details in Clinical Summary). Argatroban infusion was started on day 9 after MVR surgery, with a target value of 1.0 ng/mL. For redo surgery, heparin was administered because of negative HIT tests. For this period, argatroban was discontinued and thereafter resumed.

Two patients underwent mitral valve replacement (MVR) and endocardial cryoablation of the left atrium (CryoICE, AtriCure). Patient A was 58 years old and patient B was 61 years old. Both were male. Patient A had combined mitral valve stenosis and regurgitation, and patient B had mitral valve endocarditis. Both had chronic atrial fibrillation and enlarged left atria. Left ventricular ejection fraction was slightly reduced in patient A and preserved in patient B. Thromboembolic prophylaxis with intravenous unfractioned heparin was started in both patients on the day of hospital admission and continued until surgery with a target activated partial thromboplastin time of 60 to 80 seconds because of underlying atrial fibrillation ( Figs. 1 and 2 ).

In both patients, intraoperative repair of the mitral valve was not successful; therefore, a bioprosthesis was implanted. Patient A had concomitant coronary artery bypass grafting, and patient B had concomitant closure of a patent foramen ovale over a minimally invasive approach. Anticoagulation for cardiopulmonary bypass (CPB) during the operation was achieved using intravenous heparin. Treatment was started with an initial dose of 30 IU/kg with a target activated clotting time (ACT) of over 400 seconds.

After surgery, patient A could be weaned from the respirator within 15 hours and was transferred to the regular ward on postoperative day 6. In patient B, postoperative lung function was severely compromised and percutaneous tracheostomy was performed 2 weeks after surgery. Thereafter, weaning from the respirator was initiated but failed due to aspiration. The patient had to be sedated again and ventilated in a controlled manner. In both patients, postoperative thromboembolic prophylaxis was initially continued with unfractioned intravenous heparin.

A significant drop in platelet count was observed on day 14 (patient A) and day 9 (patient B) after the initial intravenous heparin exposure ( Figs. 1 and 2 ). Directly thereafter, heparin was replaced by argatroban (with an initial infusion rate of 0.5 µg/kg/min), using therapeutic drug monitoring with a target range in blood serum of 1.0 µg/mL. An antiplatelet factor 4-heparin (anti-PF4-H) antibody test, based on a chemiluminescent assay (ACL, AcuStar), revealed positive results for both patients (2.44 U/mL in patient A, and 4.6 U/mL in patient B, cut-off value: ≥1.0 U/mL). The chemiluminescence test is specifically designed for the detection of very low concentrations of analytes. Having a quick turnaround time of approximately 30 minutes, it has superior range and sensitivity qualities compared with enzyme-linked immunosorbent assays (ELISA) or immunoturbidimetric assays. 1 With chemiluminescent assays, the tracer binds specifically to the target of the assay, emitting light when an oxidizer and a catalyst are added. The light detector in the ACL AcuStar is highly sensitive and detects extremely low levels of light, significantly increasing the linearity (or working range) versus typical colorimetric assays. 2

The results were confirmed by two further assays, performed in the Institute for Immunology and Transfusion Medicine in Greifswald, which constitutes our reference laboratory. The tests used were a PF4-H IgG-specific ELISA 3 and a heparin-induced platelet aggregation (HIPA) test. 4 The optical density (OD) value in the ELISA test was 2.09 OD in patient A and 2.04 OD in patient B (cut-off: >1.0 OD). The HIPA test was also positive in both patients (4 of 4 cells).

In parallel to the typical serological configuration of heparin-induced thrombocytopenia type II (HIT type II), thrombotic complications and a clinical deterioration with acute hemodynamic and respiratory instability were observed for 10 days (patient A) and 5 weeks (patient B) after surgery. Therefore, patient A was an example for an early thrombotic complication in association with an HIT type II, whereas the thrombotic complication occurred late in patient B. In patient B, although heparin had been discontinued for more than 4 weeks, the thrombotic complication impressed similar to the acute reaction, which is usually observed closer to the serological diagnosis of an HIT type II or may even proceed it. 5

**Fig. 3:**
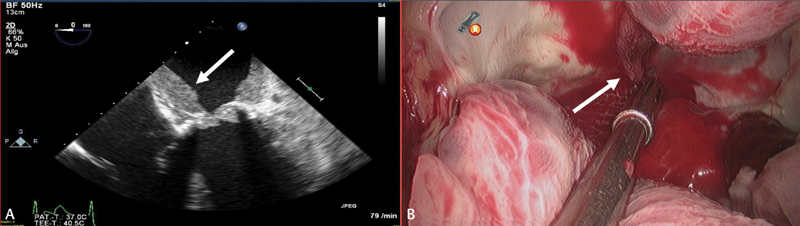
( **A** ) Massive thrombus formation ( *arrow* ) in the left atrium in transesophageal echocardiographic view. ( **B** ) Intraoperative view of thrombus sticking to imprints of lines ( *arrow* ) drawn with the cryoablation probe in the left atrium.

In both patients, echocardiography showed massive thrombus formation in the left atrium, obstructing opening of the bioprosthetic valves ( Fig. 3A ). On day 46 after initial heparin exposure, patient B was screened again for PF4-H IgG antibodies, revealing only weak positive result (OD 0.81, cut-off: > 1.0 OD) in the ELISA assay and a negative result in the HIPA test.

Both patients underwent urgent redo surgery, which could not be postponed due to the instable cardiorespiratory situation. During CPB, anticoagulation was performed with argatroban (initial infusion rate of 2 µg/kg/min), with a target ACT of over 400 seconds in patient A, and heparin (initial dose of 30 IE/kg), with a target ACT of over 400 seconds in patient B, because the HIPA test was negative in the meantime. However, after surgery, he was again treated with argatroban.

In patient A, the bioprosthesis was immured by thrombus, extending into all four pulmonary veins. Thrombus sticking to left atrial wall showed imprints of lines drawn with the cryoablation probe ( Fig. 3B ). All thrombus material was removed and the bioprosthesis was cleaned and could be left in place.

In patient B, thrombus did not only almost completely fill the left atrium but also extend into the left ventricle. The bioprosthesis had to be replaced and the left ventricle was evacuated from thrombus via the aortic valve.

Patient A developed fulminant liver failure which affected argatroban metabolization; the patient died due to uncontrolled bleeding the day after surgery. Patient B died 5 days after redo surgery of multiorgan failure. Although it was only applied perioperatively, heparin may have reactivated antibodies and triggered multiorgan failure in association with the HIT type II.

Thus, both patients died of HIT-associated complications.

## Discussion

Venous and arterial thrombus formation are well-known complications of HIT type II, 6 but reports about atrial or ventricular thrombus and prosthetic valve thrombosis after cardiac surgery are scarce. 7 8 9 About 25 to 50% of patients after cardiac surgery reveal antibodies to PF4-H complexes in peripheral blood, but only 1 to 3% develop clinical signs of HIT type II. 10 It is well known that MVR alone can result in thrombus formation in the left atrium in up to 26% of patients with otherwise uneventful postoperative courses. In the majority of patients, thrombus will regress spontaneously or under anticoagulation therapy. 11 Endocardial cryoablation itself carries the risk of thrombus formation, 12 and an increased left atrial volume, among others, is recognized as risk factor for atrial thrombus formation. 13 HIT type II has been described to trigger multiorgan failure in a cardiac surgical patient. 14

We conclude that an HIT type II triggered massive intracardial thrombus formation in two patients who had undergone MVR and endocardial cryoablation. In one patient, it occurred early at day 17, and in another one it occurred late at day 46 after initial heparin exposure. Both patients died from the HIT type II–associated complications of redo surgery.
